# miR‐126 reduces trastuzumab resistance by targeting PIK3R2 and regulating AKT/mTOR pathway in breast cancer cells

**DOI:** 10.1111/jcmm.15396

**Published:** 2020-05-15

**Authors:** Rao Fu, Jing‐Shan Tong

**Affiliations:** ^1^ College of Chemical Engineering Northeast Electric Power University Jilin city China; ^2^ Department of Pharmacology and Chemical Biology University of Pittsburgh School of Medicine Pittsburgh PA USA

**Keywords:** breast cancer, miR‐126, PIK3R2, PIK3R2/PI3K/AKT/mTOR, trastuzumab resistance

## Abstract

MicroRNAs (miRNAs) have been found to play a key role in drug resistance. In the current study, we aimed to explore the potential role of miR‐126 in trastuzumab resistance in breast cancer cells. We found that the trastuzumab‐resistant cell lines SKBR3/TR and BT474/TR had low expression of miR‐126 and increased ability to migrate and invade. The resistance, invasion and mobilization abilities of the cells resistant to trastuzumab were reduced by ectopic expression of miR‐126 mimics. In comparison, inhibition of miR‐126 in SKBR3 parental cells had the opposite effect of an increased resistance to trastuzumab as well as invasion and migration. It was also found that miR‐126 directly targets PIK3R2 in breast cancer cells. PIK3R2‐knockdown cells showed decreased resistance to trastuzumab, while overexpression of PIK3R2 increased trastuzumab resistance. In addition, our finding showed that overexpression of miR‐126 reduced resistance to trastuzumab in the trastuzumab‐resistant cells and that inhibition of the PIK3R2/PI3K/AKT/mTOR signalling pathway was involved in this effect. SKBR3/TR cells also showed increased sensitivity to trastuzumab mediated by miR‐126 in vivo. In conclusion, the above findings demonstrated that overexpression of miR‐126 or down‐regulation of its target gene may be a potential approach to overcome trastuzumab resistance in breast cancer cells.

## 1 INTRODUCTION

Breast cancer is a ubiquitous malignant disease that ranks second in mortality of women worldwide.^1^, ^2^, ^3^ Research on therapeutic interventions over the past few years has revolved around characterizing several vital signalling pathways in terms of their functions, expression and regulation.^4^ This research has improved our understanding of several subtypes of the disease that possess different biological identities.^5^, ^6^ A targeted therapy that received much spotlight is the use of anti‐human epidermal growth factor receptor 2 (HER2), as this receptor is overexpressed in several breast cancers.^7^, ^8^, ^9^ Trastuzumab is an agent used in chemotherapy that is efficient, easy to administer and has milder adverse effects.^10^, ^11^, ^12^ However, trastuzumab resistance often occurs in clinical treatment.^13^, ^14^, ^15^ Several mechanisms leading to trastuzumab resistance were proposed, including through direct or alternative pathways (such as abnormally expressed EGFR family and its ligands) and loss of PTEN function leading to excessive activation of PI3K/AKT pathway.^16^, ^17^ Therefore, novel therapy methods to overcome resistance to trastuzumab are an urgent strategy.

Recent research has revealed the role of microRNA (miRNA) expression modulation in the resistance of cancer cells.^18^, ^19^ miRNAs are a collection of small non‐coding RNAs that cause cleavage of target mRNA or prevent its translation by the potential binding between the miRNA and the 3'‐UTR of the target gene.^20^, ^21^ One such miRNA is miR‐126, which has been the patient of extensive study and characterization.^22^, ^23^ Research has been active in examining the role of this molecule in cancer as its levels are decreased in several cancers.^24^, ^25^ For instance, the migration and invasion of breast cancer cells are inhibited by miR‐126 by regulating the PI3K/AKT signalling pathway.^26^ The migration and cell death of laryngeal cancer cells are regulated by miR‐126 through VEGF inhibition.^27^, ^28^ The role of miR‐126 in human breast cancer trastuzumab resistance is not yet clear. It is reported that p85β encoded by the PIK3R2 gene is the main subtype of the PI3K regulatory subunit, while the PI3K/AKT signalling pathway can be activated by PIK3R2.^29^, ^30^ However, the role of PIK3R2 in trastuzumab resistance remains unclear.

In the current study, down‐regulation of miR‐126 was observed in trastuzumab‐resistant cells. The sensitivity to trastuzumab of these resistant cells was increased by overexpressing miR‐126 or knocking down its target gene PIK3R2. We report that the mechanism of resistance to trastuzumab by reduced miR‐126 partially involves inactivation of the PIK3R2/PI3K/AKT/mTOR signalling pathway. These results highlight novel aspects of miR‐126 and trastuzumab resistance in breast cancer that are suggestive of the use of this microRNA or its downstream targets as potential new therapeutic targets to turn the tide against resistance.

## 2 MATERIALS AND METHODS

### 2.1 Cell culture and reagents

The human breast cancer cell lines SKBR3 and BT474 were obtained from American Type Culture Collection (ATCC, Manassas, VA, USA). RPMI‐1640 supplemented with 10% foetal bovine serum (FBS, Gibco, Gaithersburg, MD, USA) was used for cell culture at 37°C and a humidified atmosphere with 5% CO_2_. Trastuzumab resistance was induced by administration of the drug at a low concentration of 5 μg/mL continuously over 6 months to SKBR3 and BT474 cells as previous study.^31^ PI828 and rapamycin were sourced from Selleckchem (Houston, TX, USA).

### 2.2 Western blotting

Western blotting was performed as previously described^32^, ^33^ with primary antibodies against phospho‐AKT (S473), phospho‐mTOR (S2448), PIK3R2, AKT, mTOR and β‐actin (Cell Signaling Technology). Briefly, indicated cells were lysed by RIPA buffer (Sigma Aldrich), and lysates containing total protein were obtained. The protein concentrations were measured using the BCA method (Sigma) and used to estimate the volume of protein yielding 50 μg, which was then subjected to gel electrophoresis (SDS‐PAGE), and transferred onto polyvinylidene fluoride (PVDF) membranes. The membrane was blocked with 5% non‐fat milk in 0.5% TBS‐T buffer for 60 minutes at room temperature and then incubated overnight with the corresponding primary antibodies at 4°C. Secondary antibody conjugated with HRP (horseradish peroxidase) was added and kept at RT for 60 minutes. The immunoblot was developed with an ECL reagent.

### 2.3 Transient transfection

GenePharma was the source of mimic NC (FAM‐labelled mimic negative control), miR‐126 inhibitor, NC inhibitor, miR‐126 mimics, ex‐PIK3R2 (PIK3R2 overexpression vector, pEZ/M98/neo‐PIK3R2), PIK3R2‐shRNA (silencing vector for PIK3R2, p‐GPU6‐PIK3R2‐shRNA), shRNA control and ex‐control. Six‐well plates were used for seeding cells that were subjected to transfection with either shRNA (0.4 μg) vectors or oligonucleotides (200 pmol). Transfections were performed according to the instructions for Lipofectamine 2000 (Invitrogen, USA), and 48 hours later, further analysis was performed.

### 2.4 Quantitative real‐time PCR (qRT‐PCR)

TRIzol reagent (Invitrogen) was used to isolate total RNA. Total RNA was then subjected to spectroscopy to determine purity and concentration. Stem‐loop RT was used for mature miRNA expression, followed by real‐time PCR. The Sanger Center miRNA Registry was the source of the miR‐126 sequence that in turn was used for primer design. An ABI 7900 HT Thermal cycler was used for qRT‐PCR (40 cycles; standard operation) in triplicate. The 2^−ΔΔCt^ method was used to calculate the fold changes. The primers are list as follows: miR126: 5’‐TATGGTTGTTCTCGACTCCTTCAC‐3’ and 5’‐TCGTCTGTCGTACCGTGAGTAAT‐3’ and U6: 5’‐CTCGCTTCGGCAGCACA‐3’ and 5’‐AACGCTTCACGAATTTGCGT‐3’.

### 2.5 Luciferase reporter assay

The probable target of human miR‐126 was predicted to be PIK3R2 using the miRDB, microRNA.org and TargetScan databases for miRNA analysis. The pmirGLO3 or pGL3 luciferase reporter vector was used for cloning the predicted portion of the 3'‐UTR of PIK3R2, which was termed PIK3R2‐WT. pmirGLO3 was also used for cloning the 3'‐UTR of PIK3R2 containing a mutated sequence, which was termed PIK3R2‐MUT. The positive control (PC) was pmirGLO3 expressing the miR‐126 inhibitor sequence. The luciferase assay was performed using cotransfection of a reporter vector and miR‐126 mimics or mimic NC in SKBR3 cell lines. The dual‐luciferase reporter assay system was used to analyse the luciferase activity of cells that were harvested 48 hours post‐transfection.

### 2.6 Migration and invasion assays

Cell migration was determined by wound healing experiments, while cell invasion was assessed by transwell insert assays. The former involved the use of a pipette tip to wound cells grown to confluence. Images were obtained at zero time and time intervals to determine the wound width as a way to assess migration. The invasion assay involved the use of the lower portion of invasion inserts coated with Matrigel. Cells in serum‐free media were added to the upper chamber, and invasion towards medium with 10% FBS was assessed after 24 hours at 37°C. The cells that invaded the membrane were fixed, stained with crystal violet and imaged to be counted using ImageJ software. All the above assays were performed in triplicate.

### 2.7 Assay for drug sensitivity in vitro

Six‐well plates were used for seeding 3 × 10^5^ cells/well with which transfection was performed. SKBR3 cells were transfected with inhibitor negative control or miR‐126 inhibitor, while SKBR3/TR cells were transfected with negative control or miR‐126 mimic. The transfection was performed with Lipofectamine 2000 (Invitrogen) by following the manufacturer's instructions. Then, 96‐well plates were used to seed 5 × 10^3^ cells/well 24 hours following the transfection. An MTS (3‐(4,5‐dimehylthiazol‐2‐yl)‐2,5‐diphenyl‐tetrazolium bromide) assay followed by measurement of A490 (absorbance at 490 nm) was used for the cell viability study 48 hours after drug treatment. A relative survival curve was used to estimate the IC50 or drug concentration that caused 50% growth inhibition. Assays were conducted in duplicate as 3 independent experiments.

### 2.8 Xenograft model

Nude mice received subcutaneous injections of 5 × 10^5^ SKBR3/TR cells in 100 μL phosphate‐buffered saline in the right flank. Seven days after inoculation with cells, 24 mice were randomly assigned into 4 groups (n = 6). Xenografts received miR‐126 agomirs and miR‐126 agomir negative control (2 nmol) every two days by direct local injection. Five milligrams/kg trastuzumab was administered every other day using intraperitoneal injection. The mice were killed at day 30 post‐treatment; the tumours were removed and weighed. Western blotting was used for the protein profile of the tumours.

### 2.9 Statistical analysis

Prism V was used for all statistical analyses, and data are presented as the mean ± SD. Student's *t* test and one‐way analysis of variance (ANOVA) were used to analyse statistical significance among groups with *P* < 0.05 considered statistically significant.

## 3 RESULTS

### 3.1 Down‐regulated miR‐126 detected in breast cancer cells resistant to trastuzumab

**FIGURE 1:**
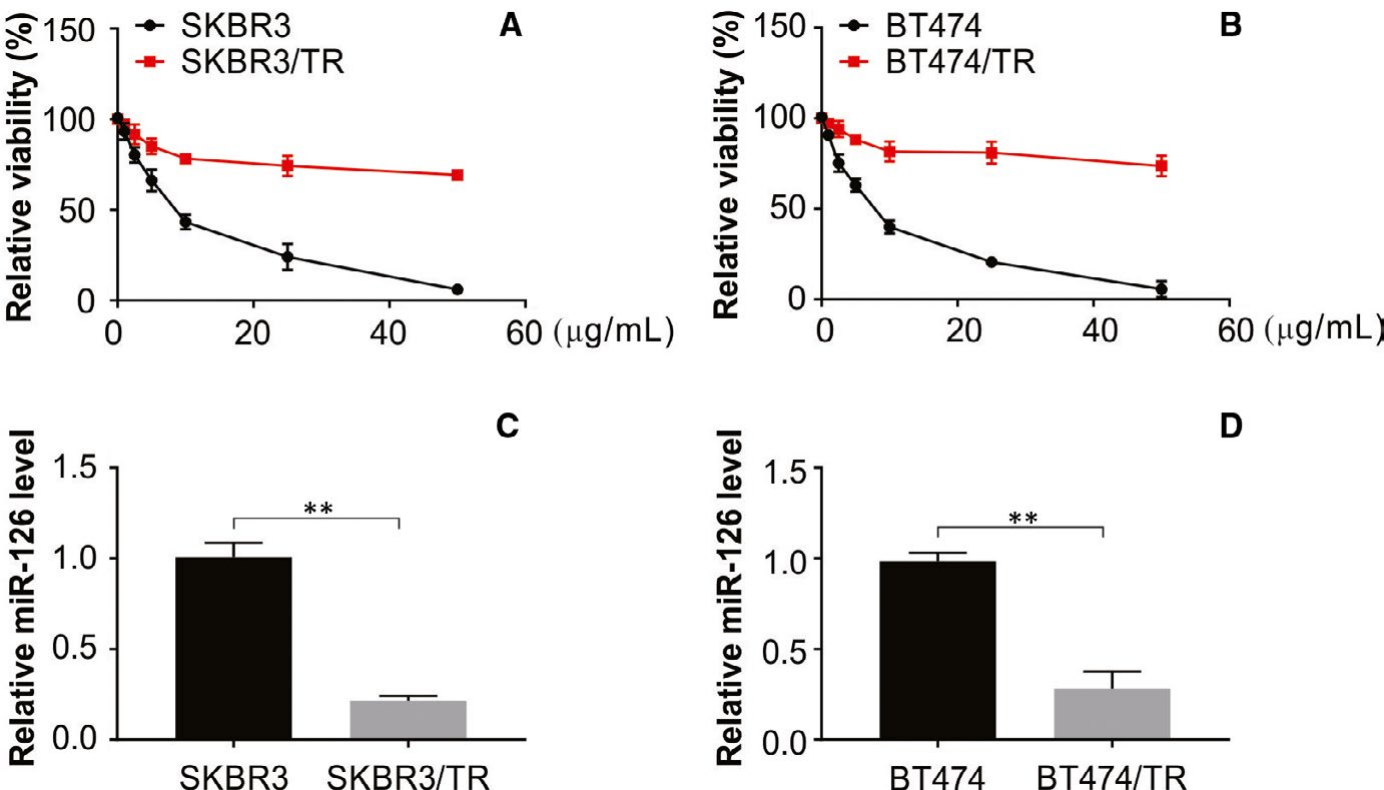
Decreased level of miR‐126 in trastuzumab‐resistant cells. (A) and (B) Two trastuzumab‐resistant cell lines and their parental cells were treated with the indicated concentrations of trastuzumab for 72 h and then were subjected to an MTS assay. (C) and (D) Real‐time RT‐PCR was used to analysed the level of miR‐126 in indicated cells. All data are mean ± SD of three separate experiments. **P < 0.01

Resistance to drugs has been shown to be an outcome of increased invasive ability caused by drugs used in chemotherapy.^34^, ^35^ To assess resistance to trastuzumab, sensitivity to the drug was compared between the resistant cell lines SKBR3/TR and BT474/TR and their parental cells. Trastuzumab resistance of the resistant cell lines was significantly higher than that of the parental cells (Figure 1A and B). Our findings showed that the IC50 values of trastuzumab were higher in SKBR3/TR cells than in SKBR3 cells and were also higher in BT474/TR cells than in BT474 cells, as shown by the MTS assay. The potential role of miR‐126 in drug resistance was assessed using the expression levels of the miRNA in the parental cell lines and resistant lines. The level of miR‐126 was significantly reduced in the resistant cell lines as shown by real‐time PCR compared with the parental cells (Figure 1C and D). The above results indicated that miR‐126 has a potential role in trastuzumab resistance in breast cancer cells.

### 3.2 Overexpression of miR‐126 reverses resistance as well as invasion and migration in trastuzumab‐resistant cells

**FIGURE 2:**
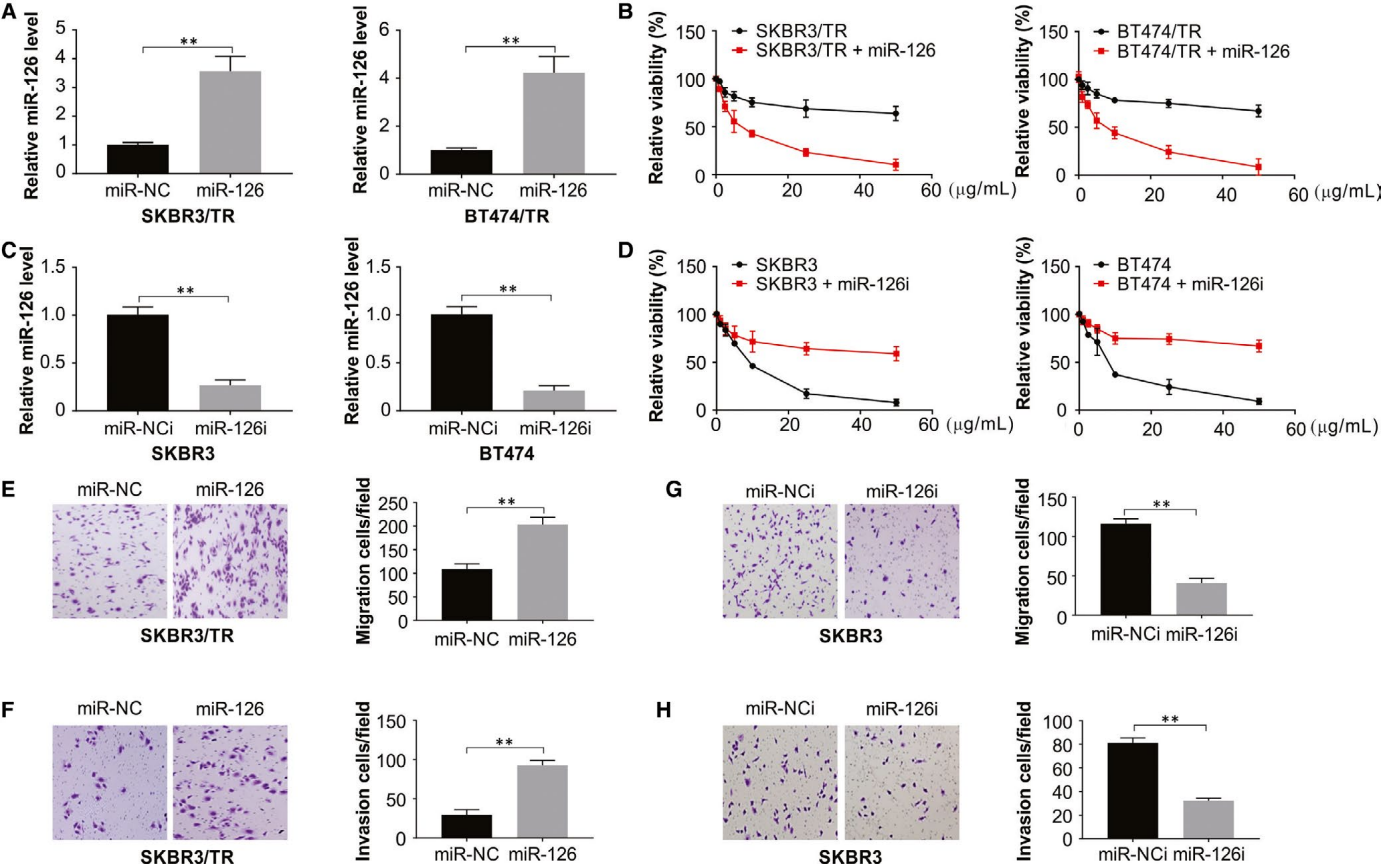
miR‐126 decreased trastuzumab resistance, migration and invasion in the resistance cells. (A) SKBR3/TR and BT474/TR cells were transfected with the miR‐126 mimic. After 24 h of transfection, miR‐126 level was detected by real‐time RT‐PCR. (B) SKBR3/TR and BT474/TR cells transfected with the miR‐126 mimic were treated with the indicated concentration of trastuzumab for 72 h and were subsequently subjected to an MTS assay. (C) SKBR3 and BT474 cells were transfected with miR‐126 inhibitor, miR‐126 level was detected by real‐time RT‐PCR. (D) SKBR3 and BT474 cells transfected with miR‐126 inhibitor were treated with the indicated concentration of trastuzumab for 72 h and subsequently subjected to an MTS assay. (E) Migration of the miR‐126 mimic transfected in SKBR3/TR cells. (F) Invasion of the miR‐126 mimic transfected in SKBR3/TR cells. (G) Migration of the miR‐126 inhibitor transfected in SKBR3 cells. (H) Invasion of the miR‐126 inhibitor transfected in SKBR3 cells. All data are mean ± SD of three separate experiments. **P < 0.01

To obtain a clearer picture of the role of the microRNA in resistance, the resistant cell lines SKBR3/TR and BT474/TR were transfected with miR‐126 mimics, while the parental cells were transfected with miR‐126 inhibitor. The use of mimics attenuated the trastuzumab resistance of SKBR3/TR and BT474/TR cells, while the use of miR‐126 inhibitor in SKBR3 and BT474 cells caused increased trastuzumab resistance (Figure 2A‐D). The migration and invasion assays showed that the abilities of SKBR3/TR cells to invade and migrate were compromised by the mimics (Figure 2E and F). In contrast, SKBR3 cells treated with the miRNA inhibitor had increased abilities (Figure 2G and H). These observations highlight the role of miR‐126 in cells resistant to trastuzumab in terms of resistance and their abilities to migrate and invade.

### 3.3 miR‐126 targets PIK3R2 in breast cancer cells

**FIGURE 3:**
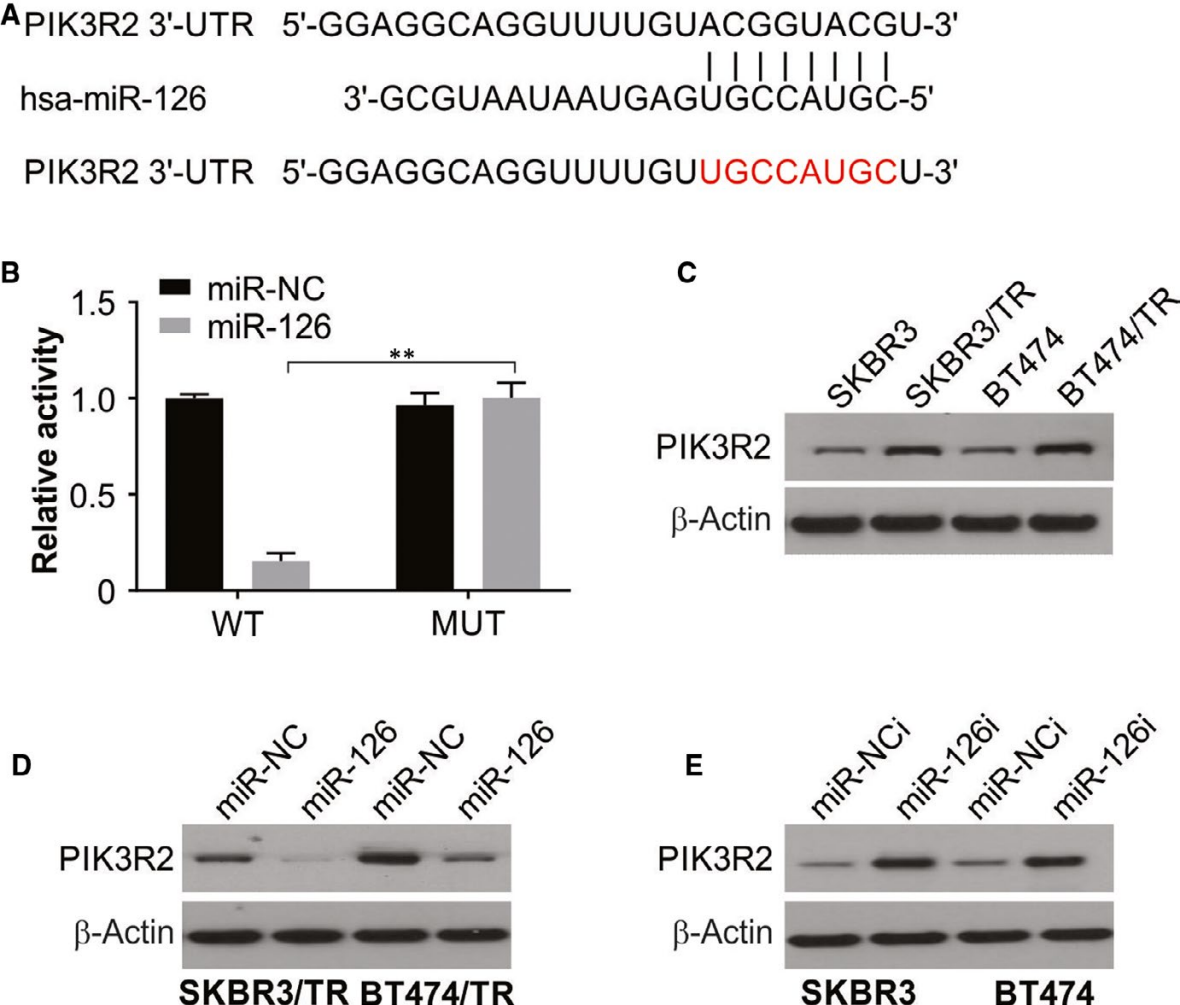
PIK3R2 is a direct target of miR‐126. (A) The predicted base pairing in miR‐126 and PIK3R2. (B) Luciferase assay was performed in SKBR3 cells that were cotransfected with the miRNA mimic and reporter vectors carrying the PIK3R2 3’‐UTR wild‐type (PIK3R2‐WT), PIK3R2 3’‐UTR mutated type (PIK3R2‐MUT). (C) PIK3R2 expression in SKBR3 and SKBR3/TR and BT474 and BT474/TR was obtained by Western blotting. (D) SKBR3/TR and BT474/TR cells were transfected with the miR‐126 mimic for 24 h. Western blotting was used to detect PIK3R2 expression. (E) SKBR3 and BT474 cells were transfected with the miR‐126 inhibitor for 24 h. Western blotting was used to detect PIK3R2 expression. All data are mean ± SD of three separate experiments. **P < 0.01

The functions of miRNAs are elucidated with analyses of the genes they target. PIK3R2 was identified as a putative target of miR‐126 using target prediction programs. The 3’‐UTR of PIK3R2 was cloned (either the wild‐type: PIK3R2‐WT or a mutated sequence: PIK3R2‐MUT) downstream of the luciferase reporter gene to assess the role of the binding site that was predicted to be in the 3’‐UTR of the target gene (Figure 3A). Cotransfection of these constructs was performed in SKBR3 cells along with miR‐126 mimics. SKBR3 cells transfected with miR‐126 had a significantly lower luciferase activity. However, no significant change in luciferase activity was observed for the PIK3R2‐MUT vector in SKBR3 cells (Figure 3B). Thus, our findings indicated a direct interaction of the 3’‐UTR of PIK3R2 with miR‐126. Interestingly, the protein level of PIK3R2 was higher in SKBR3/TR and BT474/TR cells relative to their parental cells (Figure 3C). As shown in Figure 3D and E, overexpression of miR‐126 reduced the levels of PIK3R2 in SKBR3/TR and BT474/TR cells, while down‐regulation of miR‐126 caused an increase in the levels of PIK3R2 in SKBR3 and BT474 cells. This is a confirmation of the inverse relationship between the expression of miR‐126 and PIK3R2 in these cell lines.

### 3.4 PIK3R2 mediates trastuzumab resistance

**FIGURE 4:**
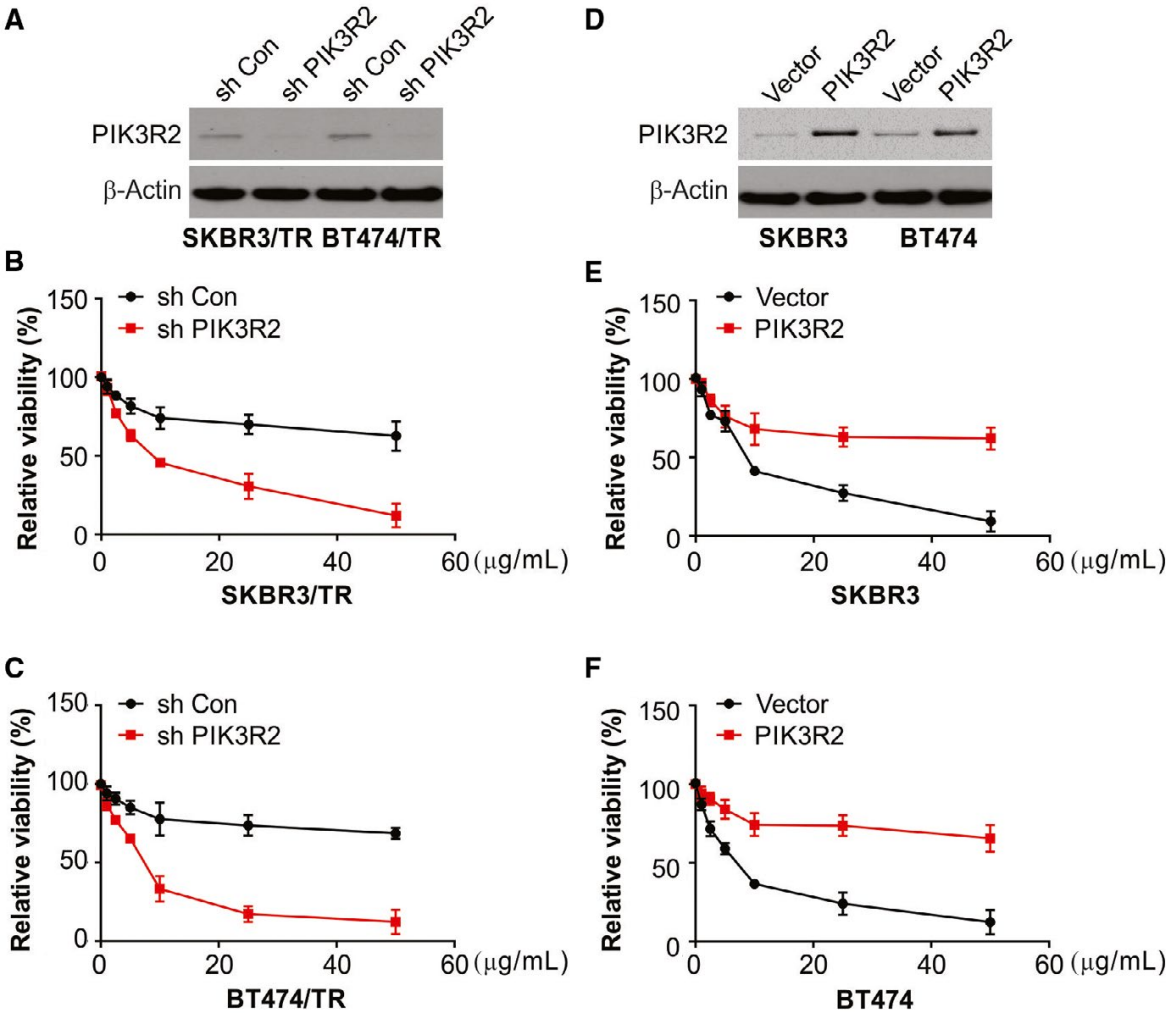
PIK3R2 is involved in miR‐126 inhibited trastuzumab resistance. (A)‐(C) SKBR3/TR and BT474/TR cells were transfected with PIK3R2 shRNA for 72 h. Relative cell viability was analysed by MTS. (D)‐(F) SKBR3 and BT474 cells were transfected with PIK3R2 expression vector (PIK3R2) for 72 h. Relative cell viability was analysed by MTS

The next step was analysis of the abilities of cells to respond to trastuzumab by using silencing and overexpression of PIK3R2. Trastuzumab sensitivity was enhanced in SKBR3/TR and BT474/TR cells when PIK3R2 was knocked down (Figure 4A‐C). However, overexpression of PIK3R2 boosted the same abilities in SKBR3 and BT474 cells (Figure 4D‐F). Therefore, the above results demonstrated that PIK3R2 mediated trastuzumab resistance in breast cancer cells.

### 3.5 miR‐126 inhibits resistance to trastuzumab through PIK3R2‐dependent PI3K/AKT/mTOR signalling activation

**FIGURE 5:**
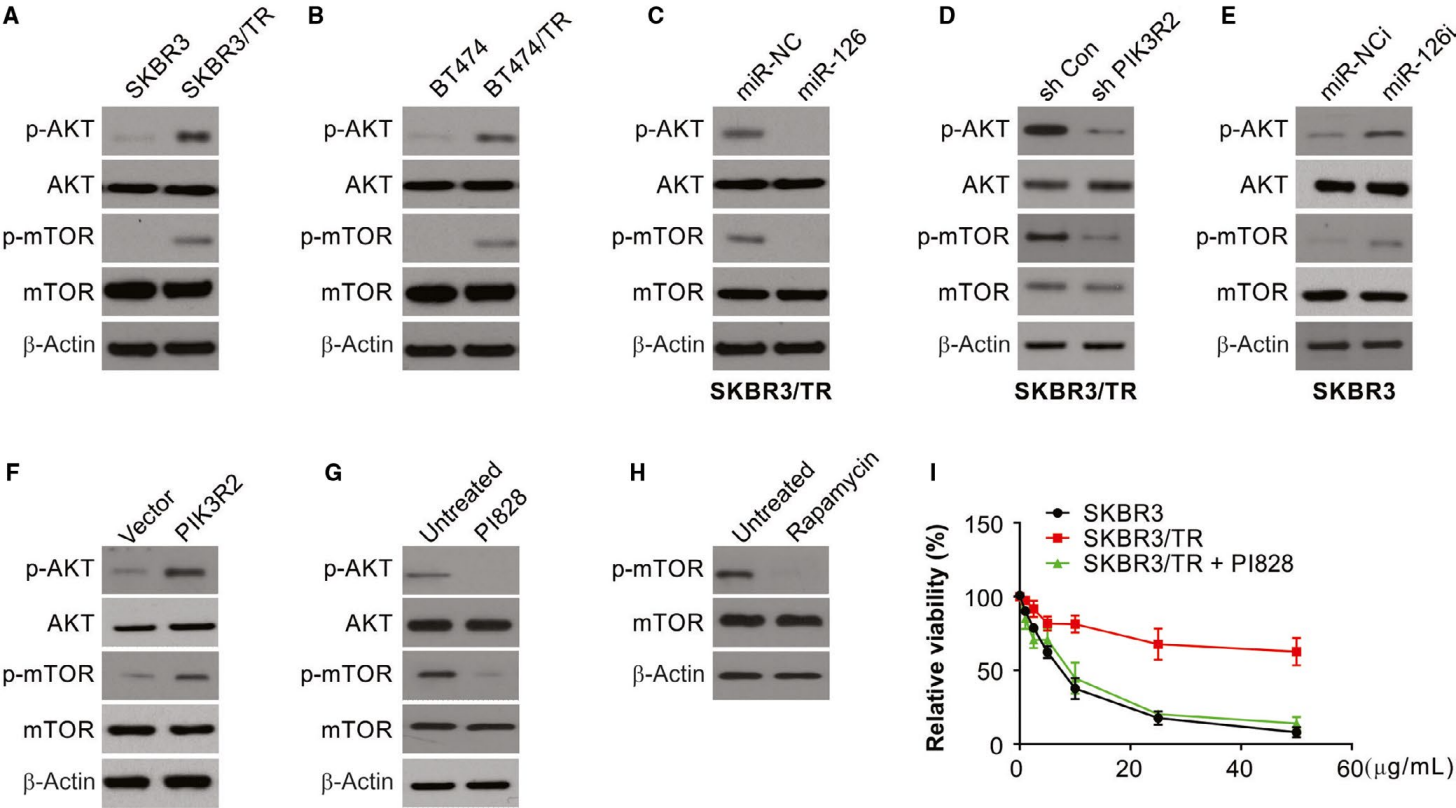
miR‐126/PIK3R2 axis regulates PI3K/AKT/mTOR signalling pathway. (A) Western blotting was performed to detect the protein expression of AKT, p‐AKT, mTOR, p‐mTOR in SKBR3/TR cells. (B) Western blotting was performed to detect the protein expression of AKT, p‐AKT, mTOR, p‐mTOR in BT474/TR cells. (C) Western blotting was performed to detect the protein expression of AKT, p‐AKT, mTOR, p‐mTOR in miR‐126 mimic‐transfected SKBR3/TR cells. (D) Western blotting was performed to detect the protein expression of AKT, p‐AKT, mTOR, p‐mTOR in PIK3R2 shRNA‐transfected SKBR3/TR cells. (E) Western blotting was performed to detect the protein expression of AKT, p‐AKT, mTOR, p‐mTOR in miR‐126 inhibitor‐transfected SKBR3 cells. (F) Western blotting was performed to detect the protein expression of AKT, p‐AKT, mTOR, p‐mTOR in PIK3R2 overexpression vectors (PIK3R2)‐transfected SKBR3 cells. (G) Western blotting was performed to detect the protein expression of AKT, p‐AKT, mTOR, p‐mTOR in PI828‐treated SKBR3/TR cells. (H) Western blotting was performed to detect the protein expression of mTOR and p‐mTOR in rapamycin‐treated SKBR3/TR cells. (I) SKBR3 and SKBR3/TR cells were treated with PI828, relative cell viability was analysed by MTS

A known target of PIK3R2 is the PI3K/AKT/mTOR pathway.^36^ Resistance to chemotherapy has been achieved efficiently by inhibiting this pathway.^37^ The activation of this pathway was shown in the resistant SKBR3/TR and BT474/TR cells (Figure 5A and B). To determine the connection between miR‐126 and the PI3K/AKT/mTOR pathway, the effects of changes in the expression of miR‐126 and PIK3R2 on the signalling pathway were investigated. SKBR3/TR cells had decreased levels of p‐AKT and p‐mTOR in the presence of miR‐126 mimics and in PIK3R2‐knockdown cells in comparison with their controls (Figure 5C and D). In contrast, the levels of p‐mTOR and p‐AKT increased in SKBR3 cells when PIK3R2 was overexpressed or miR‐126 inhibitor was used (Figure 5E and F). To ascertain the role of decreased resistance to trastuzumab by the miRNA, SKBR3/TR cells were treated with rapamycin (an inhibitor of mTOR) and PI828 (selective inhibitor of PI3K). PI828 was observed to cause a reduction in the levels of AKT and mTOR proteins, while rapamycin reduced the levels of p‐mTOR in SKBR3/TR cells (Figure 5G and H). In addition, PI828 treatment attenuated the resistance to trastuzumab in SKBR3/TR cells (Figure 5I). The above observations highlight the role of PI3K and its associated elements in the miR‐126‐induced decreased resistance to trastuzumab.

### 3.6 The in vivo sensitivity of SKBR3/TR to trastuzumab was increased by miR‐126

**FIGURE 6:**
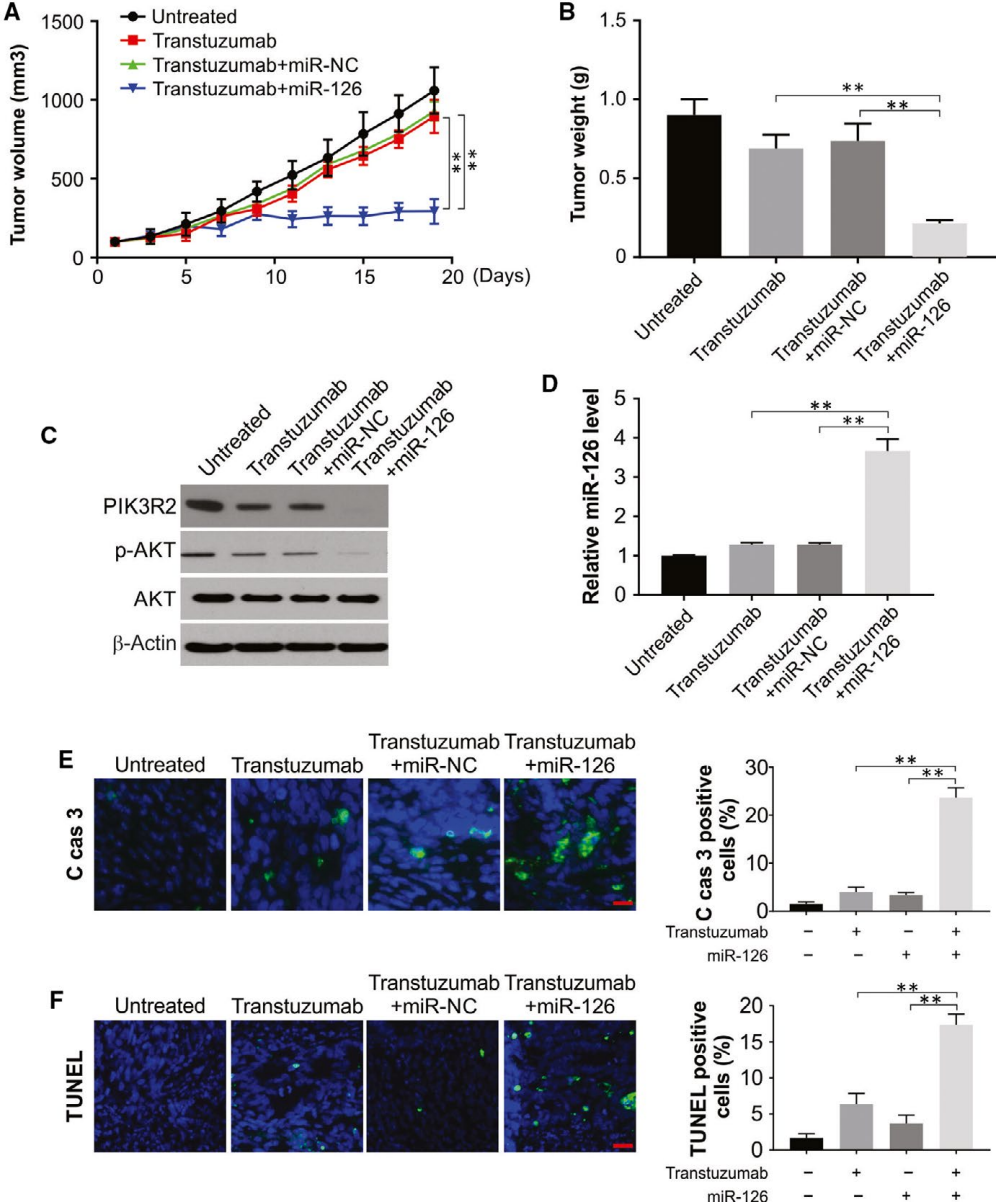
miR‐126 enhances SKBR3/TR cells to trastuzumab sensitivity in vivo. (A) Tumour volume at indicated time points. (B) The final xenograft tumour weights were measured. (C) Protein level of PIK3R2, p‐AKT in excised xenograft tumour was detected by Western blotting. (D) Real‐time RT‐PCR was used to analysed the level of miR‐126 in the tumours. (E) Paraffin‐embedded sections of tumour tissues from mice treated as indicated were analysed by cleaved caspase 3 staining. Active caspase 3‐positive cells were counted and plotted. (F) Paraffin‐embedded sections of tumour tissues from mice treated as indicated were analysed by TUNEL staining. TUNEL‐positive cells were counted and plotted. All data are mean ± SD of three separate experiments. **P < 0.01

A xenograft model was used to study the effects of miR‐126 on the sensitivity of cancer cells towards trastuzumab. Consistent with the in vitro results, the in vivo results also demonstrated that treatment with miR‐126 enhanced the sensitivity to trastuzumab in SKBR3/TR tumours (Figure 6A and B). Compared to the use of mimics or only trastuzumab treatment, the coadministration of the drug and miRNA caused significantly decreased levels of PIK3R2 as well as decreased protein levels of p‐AKT (Figure 6C and D). In addition, miR‐126 treatment also increased trastuzumab‐induced apoptosis in SKBR3/TR tumours (Figure 6E and F). The above data indicated the role of the PI3K/AKT/mTOR signalling pathway and its upstream control by miR‐126 in resistance to trastuzumab in SKBR3/TR tumours.

## 4 DISCUSSION

Among the family of human epidermal growth factor receptors (EGFR), HER2 has been linked to the activation of PI3K/AKT as well as Ras/Raf/MEK/MAPK, which has been associated with features of several cancer types in terms of the ability to divide, migrate and differentiate.^38^, ^39^ Trastuzumab, a monoclonal antibody used in therapy, specifically targets HER2.^40^ Growing research evidence points out to the roles of the compromised pathway of cell death, involvement of the glutathione pathway to detoxify drugs used in chemotherapy, efflux of these drugs using ABC or ATP‐binding cassette transporters, a reduced intake of drugs that are soluble in water and increased DNA tolerance or DNA repair that all lead to resistance to chemotherapy drugs.^41^, ^42^ A major source of resistance to trastuzumab in several tumours is the PI3K/AKT/mTOR pathway.^43^

The role of modulation of resistance to trastuzumab by miRNAs in breast cancer has been demonstrated by studies.^44^, ^45^ Our findings point to the role of such an RNA molecule, miR‐126, whose levels are decreased in the breast cancer lines SKBR3/TR and BT474/TR, contributing to resistance to trastuzumab. Reports from earlier works demonstrated the potential tumour‐suppressing activity of miR‐126 in cancers including breast cancer.^46^, ^47^ Overexpression of miR‐126 inhibited breast cancer cell invasion and metastasis. Our findings suggest a partial role for PIK3R2 and the associated PI3K/AKT/mTOR signalling as a target of miR‐126 to curb resistance to trastuzumab.

Several aspects of cancers, such as division, invasion, migration, motility and survival, involve the potential role of molecules downstream of PIK3R2.^48^, ^49^ The results of our study on breast cancer demonstrate that this network is regulated by miR‐126 and its target PIK3R2. The use of PIK3R2 shRNA and miR‐126 mimics in SKBR3/TR cells caused a decreased phosphorylation of AKT and mTOR, while the opposite effect of increased phosphorylation levels of these molecules was observed when inhibitors to miR‐126 were used or PIK3R2 was overexpressed in SKBR3 cells.

Animal studies were conducted to evaluate the anticancer effect of miR‐126 in mice that were resistant to trastuzumab. It was found that the trastuzumab sensitivity of SKBR3/TR cells was increased in the presence of this miRNA with inhibition of PIK3R2/AKT/mTOR signalling in vivo. Thus, a potential mode of addressing breast cancer resistance to trastuzumab is highlighted in this study mainly by the use of a microRNA to inhibit PIK3R2 and its associated pathway. To the best of our knowledge, this is the first study to show the role of increased levels of miR‐126 and decreased trastuzumab resistance of SKBR3 cells. To advance the study of miR‐126 and resistance to trastuzumab, research in additional cell lines is warranted, as we have conducted experiments in 2 pairs of cell lines resistant to trastuzumab.

In conclusion, inhibition of PIK3R2 and its downstream PIK3R2/AKT/mTOR signalling pathway in cells resistant to trastuzumab by overexpression of miR‐126 caused a decrease in drug resistance. This study paves the way for a possible use of miR‐126 activation or inhibition of its targets as a tool to address the resistance to trastuzumab in breast cancer cell lines.

## CONFLICT OF INTEREST

The authors declare no conflict of interest.

## Data Availability

The data that support the findings of this study are available from the corresponding author upon reasonable request.
